# Prioritisation of potential drug targets against *Bartonella bacilliformis* by an integrative *in-silico* approach

**DOI:** 10.1590/0074-02760200184

**Published:** 2020-08-10

**Authors:** Mariella Farfán-López, Abraham Espinoza-Culupú, Ruth García-de-la-Guarda, Federico Serral, Ezequiel Sosa, María Mercedes Palomino, Darío A Fernández Do Porto

**Affiliations:** 1 Universidad Nacional Mayor de San Marcos, Facultad de Ciencias Biológicas, Laboratorio de Microbiología Molecular y Biotecnología, Lima, Perú; 2 Instituto Butantan, Laboratorio de Bacteriología, São Paulo, SP, Brasil; 3 Universidad de Buenos Aires, Facultad de Ciencias Exactas y Naturales, Instituto de Cálculo, Buenos Aires, Argentina; 4 Universidad de Buenos Aires, Facultad de Ciencias Exactas y Naturales, Departamento de Química Biológica, Buenos Aires, Argentina; 5 Universidad de Buenos Aires, Facultad de Ciencias Exactas y Naturales, Instituto de Química Biológica de Ciencias Exactas y Naturales, Buenos Aires, Argentina

**Keywords:** Bartonella bacilliformis, drug targets, structurome, metabolic networks, Carrion’s disease

## Abstract

**BACKGROUND:**

Carrion’s disease (CD) is a neglected biphasic illness caused by *Bartonella bacilliformis*, a Gram-negative bacteria found in the Andean valleys. The spread of resistant strains underlines the need for novel antimicrobials against *B. bacilliformis* and related bacterial pathogens.

**OBJECTIVE:**

The main aim of this study was to integrate genomic-scale data to shortlist a set of proteins that could serve as attractive targets for new antimicrobial discovery to combat *B. bacilliformis*.

**METHODS:**

We performed a multidimensional genomic scale analysis of potential and relevant targets which includes structural druggability, metabolic analysis and essentiality criteria to select proteins with attractive features for drug discovery.

**FINDINGS:**

We shortlisted seventeen relevant proteins to develop new drugs against the causative agent of Carrion’s disease. Particularly, the protein products of *fabI*, *folA*, *aroA*, *trmFO*, *uppP* and *murE* genes, meet an important number of desirable features that make them attractive targets for new drug development. This data compendium is freely available as a web server (http://target.sbg.qb.fcen.uba.ar/).

**MAIN CONCLUSION:**

This work represents an effort to reduce the costs in the first phases of *B. bacilliformis* drug discovery.

*Bartonella bacilliformis* (Bb) is a Gram-negative pathogenic bacterium responsible for Carrion’s disease (CD) which causes haemolytic anaemia and skin lesions.^1^ This infection is endemic to some areas of Peru, Colombia, and Ecuador. It is mainly associated with poverty, climate changes, and the little financial support it receives.^2^ This disease has two different clinical phases. The early or acute phase (Oroya fever) symptoms include fever and profound anaemia and usually show up about 60 days after infection. This phase is fatal to 40-88% of patients without antibiotic intervention and even with adequate treatment, the mortality rate can increase to 9-11%. The chronic phase or Peruvian verruga is characterised by the onset of dermal eruptions known as warts. This phase is seldom fatal, but lesions can scar or bleed, and eruptions can be accompanied by fever, headache, lymphadenopathy, acute joint, and bone pains.^3^

While research has found that Bb, and another genus’ species, are highly susceptible to a wide-set of drugs *in vitro,* such as beta-lactams (including penicillins and cephalosporins), aminoglycosides and quinolones,^4^ antibiotic treatment is not always successful in patients. Several confounding factors, including patient history, frequent secondary infections, and immunosuppression elicit persistent or recurrent bartonellosis despite antibiotic treatment. For decades, Oroya fever has been treated with chloramphenicol (due to frequent intercurrent infection with *Salmonella*). Alternative drugs include beta-lactams (ampicillin, penicillin G), tetracyclines (doxycycline), macrolides (erythromycin, roxithromycin), trimethoprim-sulfamethoxazole, and fluoroquinolones (norfloxacin, ciprofloxacin).^5^ Although the second generation fluoroquinolone ciprofloxacin is the drug of choice for adults in the acute phase of Carrion’s disease, resistance to quinolones produced by mutations or substitutions of amino acids in molecular targets were reported.^6^ On the other hand, mutations conferring resistance to ciprofloxacin, rifampin, and erythromycin have been molecularly characterised.^6^ The drug of choice for treating the eruptive phase of CD is rifampin; however, it has not always been successful.^5^ Of note is that the chronic phase does not respond to either chloramphenicol or penicillin treatment.^7^

The World Health Organization (WHO) has been warning that simple infections treatment is facing an alarming crisis in the health system due to the diverse resistant mechanisms emerging and spreading globally in the last decades. Despite this critical situation, we are facing a low output antibiotic development pipeline that, coupled with regulatory challenges and unattractive costs, led to many pharmaceutical companies exiting the field. The main limitation of traditional high-throughput screening (HTS) is that only a limited set of chemicals are available in any given HTS library. As this chemical space limitation would hardly be overcome, new approaches are urgently needed to deal with the increasing problem of bacterial resistance. Nowadays, omic approaches have created new opportunities for antibiotic discovery, aiding the evaluation of protein suitability to be used as a drug target, which allows to significantly reduce the costs of the initial phases of drug discovery. Thus, the initial detection of new targets can be done entirely computationally by integrating data and applying filters to select those with better features for the later steps. An example of a filter that can be used to select potential targets would be to consider only proteins that have an inhibition site with adequate structural characteristics to bind a drug-like compound. We and others have previously used *in-silico* efforts to select drug targets in clinically relevant pathogens such as *Streptococcus pneumoniae*,^8^ *Mycobacterium tuberculosis*,^9^ *Pseudomonas aeruginosa*,^10^ *Klebsiella pneumoniae*,^11^ *Salmonella enterica* subsp,^12^ and many other pathogens. Building upon some of this type of data mining works, other researchers were able to find successful inhibitors of selected targets, such as those acting on quorum-sensing components in *P. aeruginosa* ^13^ and histidine kinases of *S. epidermidis* ^14^ and *Shigella flexneri*.^15^ While the application of computational techniques alone does not envisage a definitive identification of drug targets, this approach offers potential time saving and cost benefits by reducing the search space to candidates with increased probability of serving as targets for either new or repositioned drugs.

To our knowledge, this is the first report that uses an exhaustive application of a multidimensional data integration strategy to prioritise drug targets in Bb. Combining several layers of genomic-scale information which included genomic, metabolic, and protein structural data sources, we were able to describe a set of proteins with attractive features for target selection.

## MATERIALS AND METHODS

*Bacterial strain* - Bb USM-LMMB 07 was first isolated in 2011 by our research group in Laboratorio de Microbiología Molecular y Biotecnología - Facultad de Ciencias Biológicas - Universidad Nacional Mayor de San Marcos (UNMSM) - Lima, Perú, during an outbreak in Carmen de la Frontera district, Huancabamba province, Piura. Its genome was previously sequenced and annotated by our group.^16^ All annotations and sequences for this bacterium are available at the BioProject/NCBI (https://www.ncbi.nlm.nih.gov/assembly/GCF_001624625.1), under access number NZ_LQXX01000001.1.

*Obtaining B. bacilliformis protein sequences and preliminary analysis* - All open reading frames (ORFs) were derived from the proteome downloaded from the Uniprot database (www.uniprot.org, organism code UP000076477). All ORFs were analysed with the HMMER software (http://hmmer.org/), which assigned them to families or domains, grouped according to PFAM (https://pfam.xfam.org/) classification.

*Generation of models based on structural homology* - Since no crystallised Bb protein structures were found deposited in the Protein Data Bank (PDB) (https://www.rcsb.org/) homology-based models were built for all proteome sequences using MODELLER (https://salilab.org/modeller/) when the adequate template was available as described before.^9^ ^,^ ^11^ ^,^ ^17^ Briefly, protein sequences were PSI-BLASTed (-num_iterations 3 and -e 1e^-05^) against the UniRef50 (non-redundant sequence database, where sequences are grouped with 50% sequence identity, generated from two previous steps of clusterisation of Uniprot database). Once a position-specific scoring matrix (PSSM) was obtained, it was used to search against the PDB95 (non-redundant PDB proteins with 95% sequence identity threshold) using PSI-BLAST with E-value ≤ 10^-5^ (-e 1e^-05^) as a cutoff threshold. Up to five recovered PDB structures were used as templates for homology-based modeling using MODELLER. MODELLER was used without modifying the parameters implemented as default by the software. Five models per template for each Bb protein were built. Models were classified by the GA341 score. Only those models with GA341 above 0.7 and over 60% coverage were retained. At last, one representative model which maximises QMEAN Z-score was chosen for further analyses. To ensure the good quality of the models only those models with QMEAN-Z between - two and two were kept. With this strategy, we were able to build a total of 882 structural models from a total of 1143 ORF present in the Bb genome.

*Druggability assessment* - Druggability is a concept used to describe proteins’ ability to bind drug-like compounds, leading to protein modulation in the desired way.^17^ Druggable proteins should have a well-defined pocket with proper physicochemical features to allow binding sites prediction. Our group has developed a fast method for druggability prediction based on the open-source algorithm fpocket (http://fpocket.sourceforge.net/), which combines several physicochemical descriptors to estimate the druggability of the pockets present in proteins. Considering the drug score (DS) distribution of the protein pockets hosting a drug-like compound in the PDB, these pockets were classified into four categories: non-druggable (ND) (0.0 ≤ DS <0.2), poorly druggable (PD) (0.2 ≤ DS < 0.5), druggable (D) (0.5 ≤ DS < 0.7) and highly druggable (HD) (0.7 ≤ DS ≤ 1.0). All proteins for which structural models were obtained were subjected to this classification (see http://target.sbg.qb.fcen.uba.ar/patho/user/methodology for details).

*Off-target criteria* - All Bb USM-LMMB07 proteins were submitted to BLASTp analysis against the human proteome (NCBI assembly access GCF_000001405.36) to identify non-human-homologous targets. Hits with an E-value lower than 10^−5^ and identity of 40% or above were discarded, as they can share a high degree of structural preservation that could produce adverse effects if the bacterial protein is used as a target.

Similarly, protein inhibition of the normal flora is likely to result in adverse effects. To mitigate this possibility, Bb USM-LMMB07 proteins were compared to the proteins of the 226 representative microorganisms of human intestinal flora sequenced by the Human Microbiome Project.^18^ BLASTp assessed the number of microbiota organisms showing at least one significant match to the above-mentioned assessment criterion (E-value ≤ 10^−5^, identity ≥ 40%). This is aimed at identifying candidate proteins for which the developed drugs show an improved selectivity towards the bacterial pathogen, thus minimising the impact on the commensal gut microbiota.

*Construction of the metabolic network of Bb USM-LMMB07* - The PathoLogic module from Pathway Tools v. 20.0^19^ was used to build the Bb metabolic network (MN) using the annotated genome as input. With this tool, a Pathway/Genome (PGDB) database was created containing the predicted reactions and metabolic pathways of the organism. The PGDB database was built using organism taxonomic class = *B. bacilliformis*, NCBI taxonomy ID = 774). This metabolic reconstruction is mainly based on the EC number corresponding to each enzyme. EC number annotation has been previously performed manually during the genomic annotation of the Bb USM-LMMB07 strain (https://www.ncbi.nlm.nih.gov/assembly/GCF_001624625.1).

After manual curation of the metabolic network reconstruction was completed, it was exported in SBML format for further analysis. In particular, those reactions called “small molecules”, (i. e. reactions which involve small compounds) were exported to reduce the complexity of the network when evaluating topological measures and choke-point reactions.^20^ Once the network was exported, the frequency at which each of the metabolites appeared within the reactions was checked by using an in-house Python script. Those compounds appearing most frequently within the reactions, and not participating in the flow of carbon, were considered ubiquitous (compounds involved in a significant number of reactions), such as ATP, water and protons. These ubiquitous compounds were subsequently removed from the network because they can generate artificial interactions within the metabolism.

The resulting metabolic network was represented as a reaction graph using Cytoscape_v3.7.0,^21^ after transforming the file from the SBML format to sif (simple interaction file), using the same script mentioned above. In this representation, nodes represent reactions (i.e., usually enzymes) and there is an edge between two nodes, if a product of a reaction is used as a substrate of the reaction that follows. Graph representation allowed us to calculate choke-points and topological measures as betweenness centrality. Chokepoints are reactions that uniquely produce or consume a given product or substrate, respectively. Since it is assumed that the blocking of such types of reactions may either lead to the accumulation of a potentially toxic compound in the cell or the lack of an essential metabolite, choke-point reactions have great significance in drug targeting. The betweenness centrality of every node was calculated as described elsewhere.^17^ High values of node betweenness centrality from the metabolic perspective, reflects the participation of a reaction as an intermediary in many other transformations, and its blockage would generate disequilibrium in many different pathways.

**Fig. 1:**
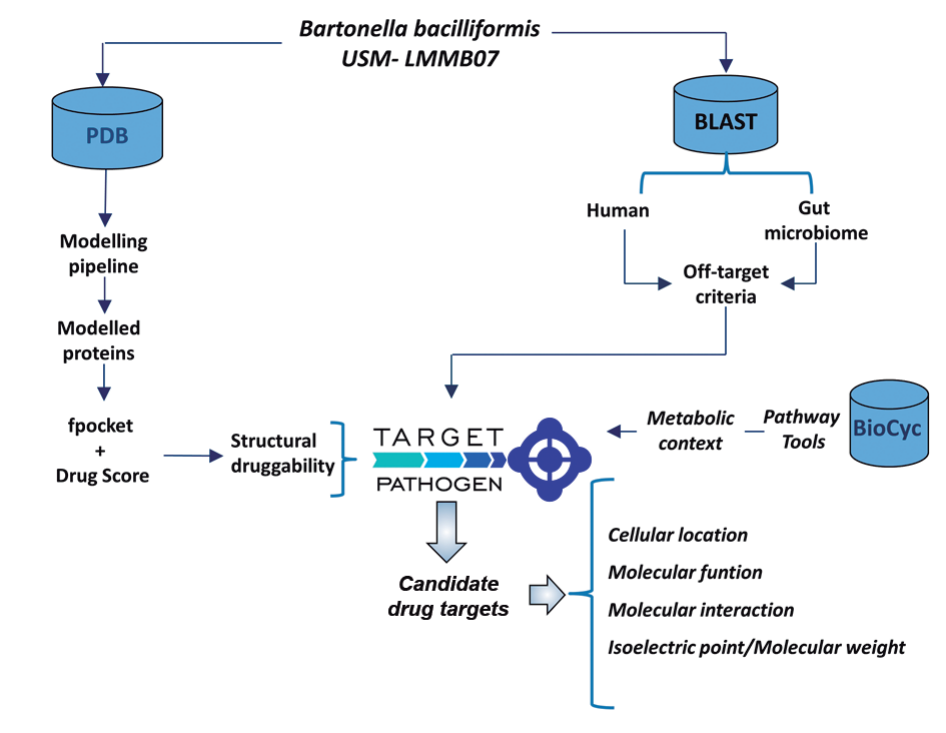
workflow for the prioritisation of molecular targets in *Bartonella bacilliformis* USM - LMMB07.

*Data integration and target prioritisation* - All previously calculated data were integrated into our own developed platform Target-Pathogen (TP),^17^ which includes a database and a web server for prioritising drug targets. Fig. 1 represents the workflow followed for molecular target prioritisation in *Bartonella*.

*Characterisation of druggable proteins for Bb USM- LMMB07* - After target prioritisation, the selected proteins were better characterised by making further analysis. PSORTbv3.0 (https://www.psort.org/), running in Bacterial- Gram-negative mode, was used to predict subcellular localisation. Gene Ontology terms were obtained from Unpriot KB. Compute pI/Mw^22^ was used to calculate the theoretical isoelectric point and molecular weight. Essentiality analysis was made by blasting the selected targets against the Database of Essential Genes (DEG) (http://www.essentialgene.org/) for homology analysis using BLASTp. The analysis was carried out against the DEG database using sequence identity ≥ 40 % and E-value ≤ 10^-5^ as the cutoff threshold. DEG proteins are classified as essential proteins based on experimental trials. If an essential homologous protein is found for a given protein, it is also likely to be essential since functions encoded by essential genes are widely conserved in microorganisms.

## RESULTS

*Bb protein structures USM-LMMB07 are enriched in druggable pockets* - Our analysis began by classifying all obtained domain structures of Bb USM-LMMB07, according to their structural druggability. For this purpose, structural domains were first grouped into two categories considering only the structural models obtained from our work scheme, since the Protein Data Bank (PDB) does not feature any information on experimentally crystallised Bb USM-LMMB07 proteins.

The first category includes structures modeled from a template that was co-crystallised in the presence of an inhibitor or a drug-like compound (MD+), thus the proteins of this group are likely to be druggable. The second category groups all modeled proteins whose structure was resolved from proteins deposited in the PDB with no drugs or associated inhibitors present (MD-).

**Fig. 2:**
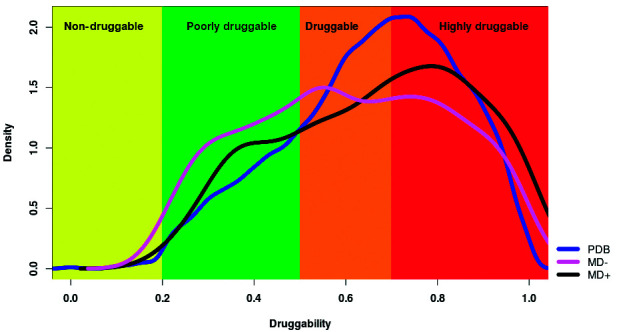
histogram of the druggability score. All Protein Data Bank (PDB) structures that bind an inhibitor or drug (blue line), only the modeled structures of *Bartonella bacilliformis* USMLMMB07 from crystallised protein structures without the presence of a drug or inhibitor (pink line) and only modeled structures of *B. bacilliformis* USM-LMMB07 from protein structures crystallised with a drug or inhibitor (black line) are represented. Considering the distribution of the score (DS) of the pockets of proteins housing a drug-like compound in the DBP, the pockets were classified into four categories: non-druggable (0.0 ≤ DS < 0.2), poorly druggable (0.2 ≤ DS < 0.5), druggable (0.5 ≤ DS < 0.7) and highly druggable (0.7 ≤ DS ≤ 1.0). The curves in Fig. 2 describe a density function in which the curves are fitted to a frequency histogram. It is important to note that in no case the druggability score is less 0 or greater than 1.

For all structures, all the possible pockets and their corresponding drug-related score (DS) were calculated using the fpocket software. Using the DS, we classified all structures in each category into four drug groups, according to the criteria set out above (Fig. 2). The results show, as expected, that most of the proteins in the MD+ group (almost 70%) have DS Scores higher than 0.5 (Table I). Thus, in agreement with previous works^9^ ^,^ ^11^ our method can predict to a high degree the likelihood of a protein to host a drug-like compound. The first group of interest, where novel targets can be found, concerns the MD+-HD group (which includes proteins in the MD+ group and are also highly druggable), the fact that both an association criteria (assignation to MD+) and a structural criteria (the DS) match is a strong argument for the selection of these 110 proteins as drug targets [Supplementary data **(Table I)**].

**TABLE I:** *Bartonella bacilliformis* USM-LMMB07 proteins classified according to druggability score (DS)

Non-druggable	MD+	MD-	Total
	18 (7.46%)	96 (14.97%)	114
Poorly druggable	55 (22.82%)	181 (28.23%)	236
Druggable	58 (24.046%)	159 (24.81%)	217
Highly druggable	110 (45.64%)	205 (31.98%)	315
Total	241	641	882

MD+: structures modeled from crystallised proteins with an inhibitor or drug; MD-: structures modeled from crystallised proteins without an inhibitor or drug.

*Reconstruction of the metabolic network of Bb USM- LMMB07* - In the next step of our prioritisation procedure, we used Pathway tools and manual revision to build a comprehensive metabolic network of Bb USM- LMMB07 from its annotated genome. A total of 898 reactions (885 catalysed by enzymes and 13 by transporters) composed the MN. These enzymatic reactions are distributed as part of 148 metabolic pathways, which groups 1143 proteins.

For comparison, our previously constructed *K. pneumoniae* metabolic network^11^ is composed of 1969 reactions with 1847 being enzyme-catalysed and forming part of 321 predicted metabolic pathways. This result correlates with both genome size.

After the construction of the metabolic network, it was exported in systems biology markup language (SBML) format for downstream analyses. Reactions involving macromolecules (such as proteins, DNA, and RNA) were filtered out, and only reactions involved in small-molecules metabolism were considered. The rationale for this strategy was that since most of the current antibiotics target macromolecules (such as ribosomes, proteins, lipids), the focus on enzymatic activities that are not related to these types of molecules would comprehend an unexplored universe suitable for drug discovery.

**Fig. 3:**
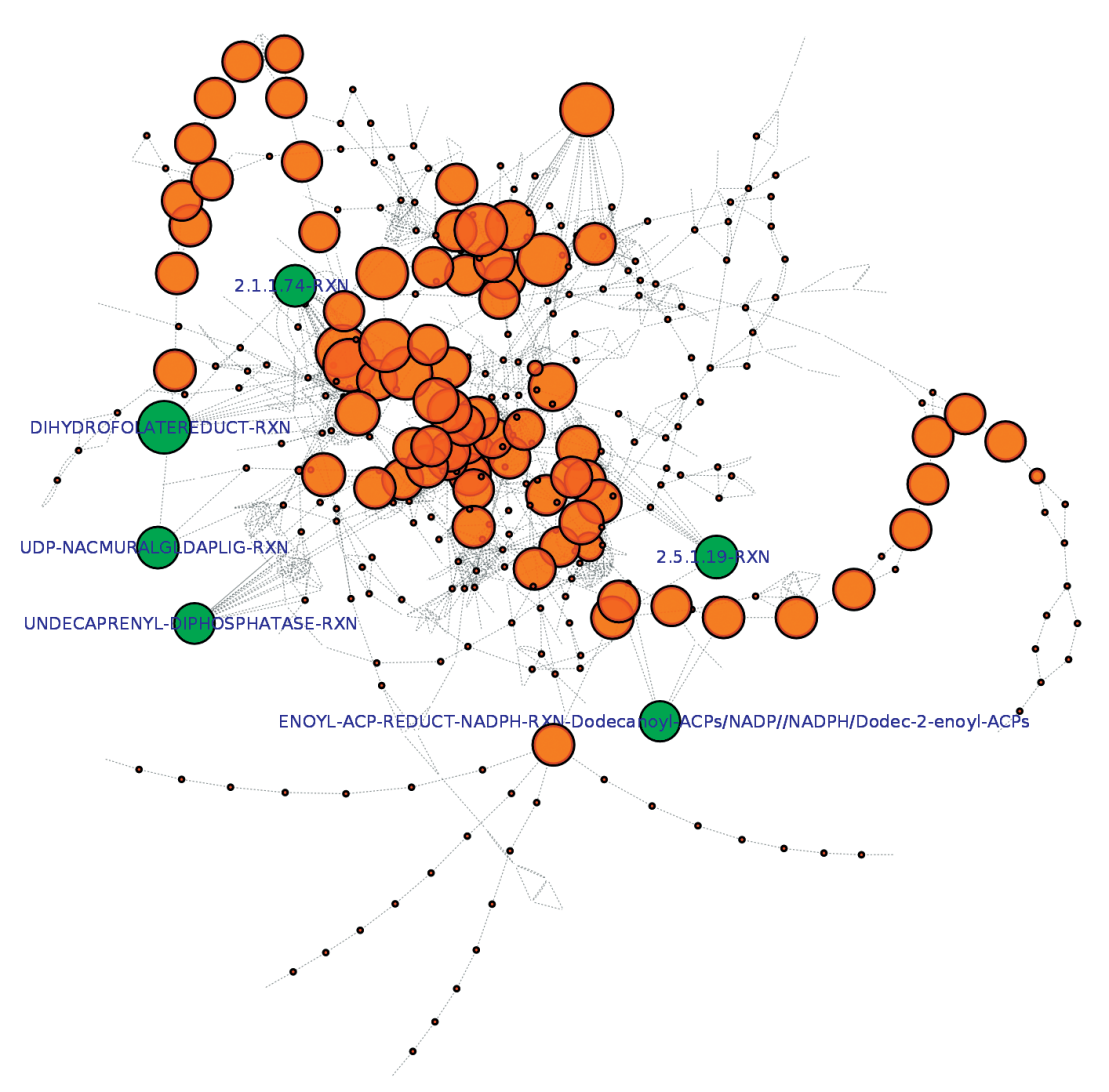
metabolic network of *Bartonella bacilliformis* USM-LMMB07 represented as a reaction graph. Nodes represent reactions in the network, and there is an edge between two nodes when the product of a reaction is used as a substrate in the next reaction. The node size is proportional to the betweenness centrality, and the MetaCyc access numbers (http://metacyc.org) are displayed for the central reactions involving the final six priority proteins.

After exporting the Bb-MN, we calculated the frequency with which all compounds were involved in the predicted reactions. The most frequent compounds were considered a potential ubiquitous compound (such as water, protons, NAD, NADH, ATP, and other cofactors). After manual inspection, a total of 33 compounds were filtered out to avoid artificial links creation on the reaction graph. Cytoscape v. 3.7.0 was then used for graphic visualisation and topological metrics calculation, particularly the betweenness centrality (BC) (Fig. 3). The presence of few high-centrality nodes indicates that these centers can be of particular importance for network cohesion. In such regard, 80 reactions were identified with a high degree of centrality (> 0.1), involving 60 proteins. On the other hand, choke-point reactions were also identified in the metabolic network of Bb USM- LMMB07, i.e. reactions that uniquely consume or produce a given compound. A total of 223 reactions were classified as consumption choke-points, while 50 reactions were production choke-points. Besides, 16 reactions were classified as double choke-point reactions (both consumption and production). Choke-point reactions blockade may lead to the lack of production of essential metabolites or potentially toxic compounds accumulation in the cell; thus, this type of reactions have great significance in drug targeting.

**Fig. 4:**
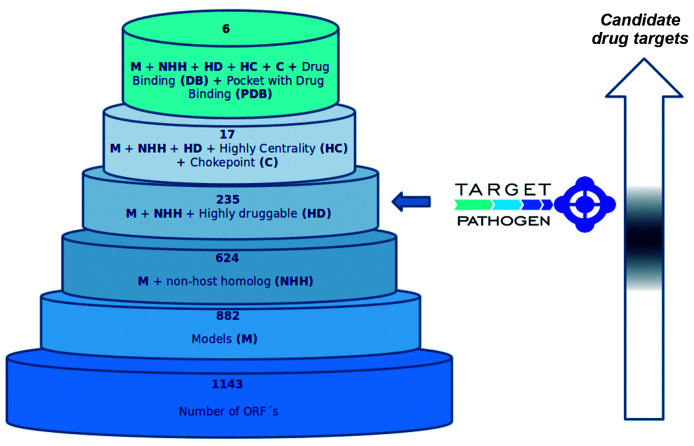
general scheme of the prioritisation process in *Bartonella bacilliformis* USMLMMB07 using Target-Pathogen filters.

*Integration of metabolic data, druggability information, and off-target criteria make it possible to identify and prioritise potential molecular targets in drug development against B. bacilliformis* - All previously calculated data was integrated into Target-Pathogen.^17^ To obtain a set of proteins that fulfill different features that make them attractive for drug discovery projects, such as structural druggability, off-targeting, essentiality, and the metabolic role we apply a set of filters (Fig. 4). First, all those proteins for which a representative structural model could not be obtained were discarded; only 882 out of the 1143 proteins making up the Bb USM-LMMB07 proteome met this requirement. Subsequently, proteins with close homologs in the human genome and at least one-third of the genomes of the gut microbiome were ruled out to minimise the chances of cross-interference (and toxicity) of a drug with human host proteins. Then, all those proteins that were not highly druggable (DS < 0.7) were filtered out and 235 highly druggable proteins with no close homologs in the human genome and its microbiome were kept [Supplementary data **(Table II)**]. Afterward, we further filter these proteins by taking into account its importance from the metabolic point of view. In this sense, we kept only those proteins involved in high centrality and choke-point reactions. The resulting 17 proteins (Table II) harbor high potential for drug targeting.

**TABLE II:** Selection of *Bartonella bacilliformis* USM-LMMB07 proteins by incorporating centrality, chokepoint and drug binding filters

Locus tag	Gene	Gene product	DS	Human off target	Gut microb	C	Chk	DB	PB
AWH67_RS01355	*rdgB*	non-canonical purine NTP pyrophosphatase	0.93	0.72	1	0.97	True	False	False
AWH67_RS04105	*folA*	dihydrofolate reductase	0.97	0.68	21	0.96	True	True	True
AWH67_RS00185	*maf2*	septum formation protein	0.85	0.64	12	0.97	True	False	False
AWH67_RS04665	*purB*	adenylosuccinate lyase	0.86	0.73	51	0.77	True	False	False
AWH67_RS05475	*-------*	beta-ketoacyl-ACP synthase II	0.97	1	0	0.21	True	False	False
AWH67_RS03970	*murE*	UDP-N-acetylmuramoyl-L-alanyl-D-glutamate--2, 6-diaminopimelate ligase	0.95	1	0	0.2	True	True	True
AWH67_RS05335	*fabZ*	beta-hydroxyacyl-ACP dehydratase	0.92	1	22	0.19	True	False	False
AWH67_RS05565	*fabH*	3-oxoacyl-ACP synthase III	0.89	1	67	0.21	True	False	False
AWH67_RS01510	*aroA*	3-phosphoshikimate 1-carboxyvinyltransferase	0.78	1	23	0.27	True	True	True
AWH67_RS02160	*trmFO*	FADH(2)-oxidising methylenetetrahydrofolate--tRNA-(uracil(54)-C(5))- methyltransferase TrmFO	0.75	1	72	0.2	True	True	True
AWH67_RS02410	*uppP*	undecaprenyl-diphosphatase	0.74	1	3	0.12	True	True	True
AWH67_RS03995	*murG*	undecaprenyl diphosphate-muramoylpentapeptide beta-N- acetylglucosaminyltransferase	0.73	1	0	0.12	True	False	False
AWH67_RS01650	*fabI*	enoyl-(acyl-carrier-protein) reductase	0.99	0.71	16	0.1	True	True	True
AWH67_RS04685	*purQ*	phosphoribosylformylglycinamidine synthase subunit	0.89	0.75	40	0.1	True	False	False
AWH67_RS01345	*dapE*	succinyl-diaminopimelate desuccinylase	0.75	0.78	11	0.2	True	False	False
AWH67_RS04690	*purL*	phosphoribosylformylglycinamidine synthase subunit	0.79	0.77	39	0.1	True	False	False
AWH67_RS02870	*fabI*	enoyl-(acyl-carrier-protein) reductase	0.77	0.73	16	0.1	True	False	False

C: centrality; Chk: chokepoint; DB: drug binding; DS: druggability score; PB: pocket with binding. -Best candidate proteins are highlighted in yellow.

**Fig. 5:**
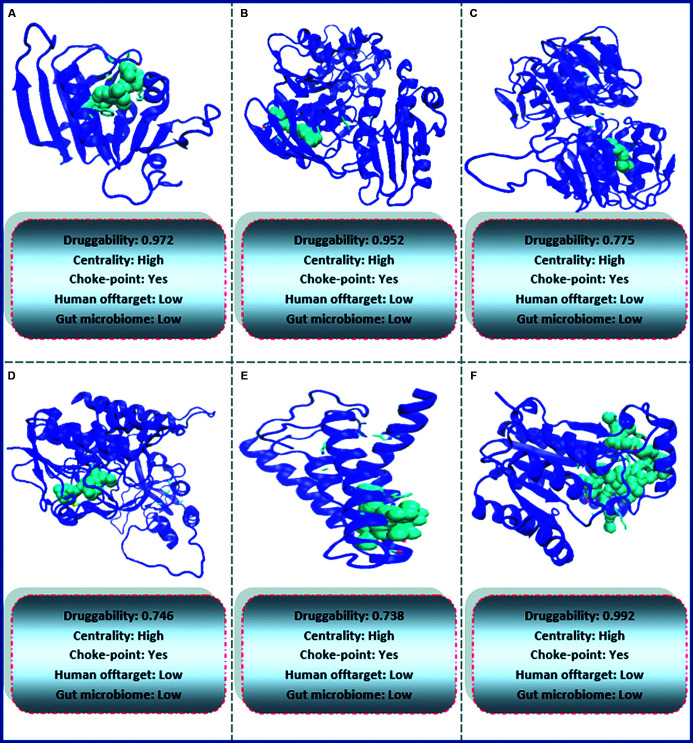
the structures correspond to the six proteins with the best characteristics to be used as possible molecular targets. Green represents the most druggable pocket in each of the proteins. (A) folA; (B) murE; (C) aroA; (D) trmFO; (E) uppP; (F) fabI.

At last, special attention was paid to those proteins for which there is at least one crystal structure, of another protein from the same PFAM domain that was crystallised in the presence of an inhibitor or drug-like compound. The proteins of this group are even more likely to be druggable since both, an association criteria (exist a protein of the same PFAM family that was co-crystallised with drugs) and a structural criteria (DS > 0.7) match. Moreover, in six (FabI, FolA, AroA, TrmFO, UppP, MurE) of a total of seven proteins that fulfill these criteria, the predicted druggable pocket overlaps with the site where the drug binds the protein in the co-crystallised structure (Fig. 5). Table III shows the six proteins that fulfill all the previously described criteria and therefore are attractive to be used as targets in drug development against *B. bacilliformis*.

**TABLE III:** Characterisation of best target proteins for *Bartonella bacilliformis* USMLMMB07

Gene name	Description	Pi*	Mw**	Subcellular localisation	Molecular function	Biological process
*fabI*	enoyl-(acyl carrier-protein) reductase	5.78	29 368.63	Cytoplasmic membrane	Catalytic activity	Fatty acid biosynthesis process
*folA*	dihydrofolate reductase	6.59	18 799.5	Cytoplasm	NADP binding activity	Metabolic process of a carbon Biosynthetic nucleotide process Biosynthetic process of amide
*aroA*	3-phosphoshikimate carboxyvinyltransferase	8.50	47 559.16	Cytoplasm	Transferase activity	It is part of the processes of the family of aromatic amino acids, of the biosynthetic process of chorismate
*trmFO*	FADH(2)-oxidising methylenetetrahydrofolate--tRNA-(uracil(54)-C(5))- methyltransferase	7.21	51 693.32	Cytoplasm	Flavin adenine dinucleotide binding methyltransferase activity	tRNA Processing
*uppP*	undecaprenyl-diphosphatase	9.2	22 650.04	Cytoplasmic membrane	Catalytic activity	Integral component of the membrane
*murE*	UDP-N-acetylmuramoyl-L- alanyl-D-glutamate--2, 6 -diaminopimelate ligase	6.51	52 537.89	Cytoplasm	Union binding drugs	Cell cycle, regulation of the cell form, biosynthetic process of peptidoglycan, cell division and organisation of the cell wall

Pi: isoelectric point; Mw: molecular mass.

*Bartonella bacilliformis targets characterisation* - Various layers of information were combined, including whole-structural, metabolic, and genomic data that allowed a shortlisting of target proteins with characteristics from a druggability standpoint. The six highlighted proteins are druggable, don’t have close homologs in the human genome, and are relevant from the metabolic point of view.

To further characterise these proteins, we analysed their essentiality, ontology (molecular function and the biological processes they perform), subcellular localisation, isoelectric point, and molecular weight (Table III).

The protein product of *fabI* is an enoyl-[acyl-carrier-protein] reductase and was found to be located in the cytoplasmic membrane. This protein develops fatty acid biosynthesis processes in the bacterium. The homology analysis through BLASTp against the DEG database shows a high degree of similarity to *Sphingomonas wittichii* RW1 and *Caulobacter crescentus* FabI (identity = 60% and 52.89, E-value = 3.63e^-109^ and E-value of 1.23e^-97^ respectively). This protein was also reported to be essential in many other bacteria such as *E. coli* and *M. tuberculosis*.^23^ In this sense, likely, Bb *fabI* is also essential since functions encoded by essential genes are broadly conserved in microorganisms. Dihydrofolate reductase (DHFR) FolA is a protein with binding NADP activity that participates in the metabolism of drugs, in the biosynthetic process of amide, and the biosynthetic process tetrahydrofolate. PSORTb analysis reveals that FolA is found in the cytoplasm. Protein search using DEG reveals that there are essential genes, homologous to Bb *folA* (identity > 40% and E-value = 1.14e-41), present in more than ten different organisms, such as *Caulobacter crescentus* and *P. aeruginosa*.

3-phosphoshikimate 1-carboxyvinyltransferase AroA; it is also located in the cytoplasm of *B. bacilliformis*. This protein intervenes in aromatic amino acids and chorismate biosynthesis as revealed by ontology analysis. High identity was found against *Rhodopseudomonas palustris* CGA009 and *C. crescentus* AroA (53.3% and 50.9% respectively). As this protein is essential in both bacteria, it may also be essential for *Bartonella*. Protein FADH(2)-oxidising methylenetetrahydrofolate--tRNA-(uracil(54)-C(5))-methyltransferase (TrmFO) is involved in tRNA processing, performs methyltransferase activity and participates in the binding of flavin adenine dinucleotide. This protein was found to be located in the cytoplasm. *Staphylococcus aureus* N315 *trmFO* (*gid*) essential gene was found to be homologous to this Bb protein (identity=48% and E-value=2.98e^-134^). Undecaprenyl-diphosphatase UppP is an integral protein of *B. bacilliformis* cytoplasmic membrane. This protein has been reported as involved in the synthesis of the carrier lipid undecaprenyl phosphate that is essential for the biosynthesis of peptidoglycan in other bacterial species. The enzyme catalyses the dephosphorylation of undecaprenyl diphosphate (UPP). The reaction product is a lipid molecule, that transports the peptidoglycan precursors from the cytoplasm to the periplasm through the cytoplasmic membrane. However, we did not identify homologous genes in DEG. The last prioritised protein is the cytoplasmic UDP-N-acetylmuramoyl-L-alanyl-D-glutamate-2, 6-diaminopimelate ligase (MurE). This protein is involved in the cell cycle, the biosynthetic process of peptidoglycan, cell division, and cell wall organisation. After performing the homology analysis against the DEG database, we noted that it presents an identity greater than 50% with the essential proteins of *Agrobacterium fabrum* str. C58 and *R. palustris* CGA009 (E-value = 0.0 and 4.54e^-146^ respectively). It is thus inferred that it may be essential for *Bartonella’s* survival.

## DISCUSSION

In this work, we make an integrative analysis framework for protein target prioritisation using as model organism *B. bacilliformis* strain USM- LMMB07, collected in 2011 in northern Perú (Huancabamba, Piura) and identified as the causal agent of Oroya fever. Different layers of omic information were combined, including genomic, structural, and metabolic data which allowed a shortlisting of targets with desirable characteristics from a druggability point of view. Out of 1143 predicted proteins that form the proteome of Bb USM- LMMB07, we obtained 882 structural models, and we could predict high druggable pockets for 235 of them. Metabolic network reconstruction of this strain allowed metabolic complement and enzymatic activities identification performed by Bb, as well as important topological metrics in the metabolic network. This information allowed us to contextualise functional aspects of the proteins identified as promising targets. All this information, along with other genomic features calculated for each protein, were loaded into an openly available web-server (Target Pathogen),^17^ that allows easy retrieval of any of the generated data, along with parameter customisation.

By applying a set of filters, we were able to delineate a unique set of six druggable proteins with no close homologs in humans (and its microbiome) and that are of interest for drug discovery. One of them, FabI participates in fatty acid synthesis (FAS) processes. The proteins of this pathway, such as FabA, FabB, FabD, FabI, and FabH, have an essential role during the biosynthesis of phospholipid membranes, lipoproteins, and LPS and represent attractive targets due to the structural differences between the human and bacterial proteins.^23^ With two commercially available inhibitors, triclosan and isoniazid (a first-line antituberculosis drug), FabI was the favorite target for antimicrobial efforts targeting FAS^24^ and was previously proposed as an attractive target to combat *K. pneumoniae*.^11^ Another druggable protein found in Bb is FolA which provides the main dihydrofolate reductase activity in the tetrahydrofolate or vitamin B9 pathway. The enzyme catalyses dihydrofolate the reduction to tetrahydrofolate via hydride transfer from NADPH to C_6_ of the pteridine ring. Tetrahydrofolate is an important intermediate in the biosynthesis of proteins and nucleic acids, it is biosynthesised *de novo* by bacteria and is involved in important biosynthesis pathways such as methionine, purines, thymidylate, and pantothenate. Humans are entirely dependent on nutritional sources of folate, transforming it into an essential vitamin. Because dihydrofolate reductase is essential for cell division and growth, it is an attractive target for drug development. Antimicrobial agents against FolA, such as 5-substituted-2, 4-diaminopyrimidine (TMP), are widely used to treat *Streptococcus pyogenes*, *S. pneumoniae*, *Escherichia coli, and K. pneumoniae* infections.^25^ Additionally, the use of sulfonamide, such as sulfamethoxazole (SMZ), a potent and selective inhibitor against another key enzyme of folate biosynthesis (dihydropteroate synthase) together with TMP, is used for certain infections treatment of since their combination causes a synergistic effect, resulting in a dramatic enhancement in antibacterial activity.^26^ The finding of FabI and FolA proteins in our work contributes towards validating our methodological approach.

The protein product of *aroA*, 3-Phosphoshikimate-1-carboxyvinyltransferase (EPSP synthase), is another target identified through our prioritisation pipeline. This protein participates in the biosynthesis of aromatic amino acids, siderophores, and metabolites such as folate, ubiquinone and vitamin K. EPSP synthase is involved in the 6th step of the chorismate pathway, catalysing the transfer of the enolpyruvoyl moiety from phosphoenolpyruvate to the hydroxyl group of carbon 5 of shikimate 3-phosphate with the phosphate elimination to produce 5-enolpyruvoyl shikimate 3-phosphate (EPSP).^27^ The shikimate pathway is present in bacteria, fungi, algae, plants, and apicomplexan parasites, but is absent in humans.^28^ Moreover, several bacterial pathogens use chorismate-derived siderophores as virulence factors. Inhibition of EPSP synthase is the basis of the widely used herbicide glyphosate. As widely reported, Class I EPSP synthases, which are present in plants and some bacteria (*e.g. Salmonella typhimurium* and *E. coli*), are inhibited at low concentrations of glyphosate.^29^ Several properties of *aroA* gene product make it attractive as an antimicrobial target, including essentiality, druggability, and the lack of a human homolog.

Our analysis also revealed methylenetetrahydrofolate-tRNA-(uracil-5-)-methyltransferase (TrmFO) as a promising target to combat Bb. TrmFO is a ﬂavin dinucleotide (FAD) binding protein, which has been identiﬁed as a tRNA modiﬁcation enzyme, responsible for the folate-dependent m5U-54 biosynthesis. In bacteria, RNA modification enzymes have been reported to exhibit a moonlighting function associated with many cell processes like virulence, stress response, morphology, growth, antibiotic susceptibility, and others.^30^ The GidA (TrmFO) tRNA modification pathway has been implicated in bacterial virulence regulation as demonstrated by diverse pathogens attenuation following the deletion of *gidA*. Furthermore, this gene deletion has produced defective cell growth, decreasing the growth by approximately 10-30% in several bacteria like *S. enterica*, *P. aeruginosa,* and *E. coli*.^31^ ^,^ ^32^ ^,^ ^33^ Given that GidA mutation has a pleiotropic effect, affecting diverse phenotypic traits, including bacterial cell growth, *gidA* could be considered a novel and potential target in *B. bacilliformis*. Another attractive target found by our analysis is Undecaprenyl-diphosphatase UppP. This protein has been reported as essential in other bacteria, however, *in vitro* analysis is required to determine its essentiality in *B. bacilliformis*. Overexpression of undecaprenyl-diphosphatase generates resistance to bacitracin.^34^ This enzyme has been studied *in-silico* as a druggable molecular target in *Streptococcus suis* ^35^ and *Clostridium botulinum*.^*36*^ We have found that this protein harbors a set of features that defines a promising target to be used for drug development for *B. bacilliformis*; regarding it as a potential target of new targeted drugs to destabilise the cell wall to disrupt bacteria growth.

The protein product of *murE*, the UDP-N-acetylmuramoyl-L-alanyl-D-glutamate-L-lysine/2,6-diaminopimelate ligase, is a bacterial enzyme belonging to the ATP-dependent Mur ligases, which are essential for peptidoglycan (PG) synthesis. PG is a key component of the cell wall of almost all eubacteria, it is responsible for rigidity and shape of bacterial cells, serves as a platform for anchoring other cell envelope components and is essential for growth and survival of bacteria. The enzymes that catalyse PG are considered one of the best sources of antibacterial targets. Similar to the other Mur class enzymes, MurE has been targeted in efforts to produce novel antimicrobials. Different chemical compounds are being studied taking into account their SAR (structural-activity relationship) studies and inhibitory potential. Such is the case of hyperenone A which is obtained from hexane and chloroform extracts of the different aerial parts of plant *Hypericum acmosepalum* and quinolone derivatives as the novel N-methyl-2-alkenyl-4-quinolone are capable to inhibit MurE ligase of *M. tuberculosis* and Methicillin-resistant *S. aureus* (MRSA).^37^

With regards to the cellular compartment where these candidates are found, most of the top-ranked proteins are located either in the cytoplasm or in the cell membrane and are a priori unavailable for external binding. This apparent inappropriateness of cytoplasmic proteins to serve as a target should not hamper their further exploration, as it is well-known that many antibiotics are capable of efficiently crossing bacterial membranes (either by diffusion or through porin channels),^38^ coupled with recent developments of delivery strategies including the use of siderophores, cyclodextrins, metal nanoparticles, antimicrobial/cell-penetrating peptides and fusogenic liposomes.^39^ Thus, the fact that a candidate target locates cytoplasmically should not detain the future design of antibacterial drugs directed towards their inhibition.

Further studies are warranted to follow-up experimentally on our elicited targets, and we invite the scientific community dedicated to this subject to help pursue these goals, thus strengthening the ongoing fight against pathogenic bacteria.
