# Supplementary material for: Prioritisation of potential drug targets against Bartonella bacilliformis by an integrative in-silico approach
Source: Mem Inst Oswaldo Cruz. 2020 Aug 10;115:e200184. doi: 10.1590/0074-02760200184 (PMC7416641; doi:10.1590/0074-02760200184)
Supplement: Supplementary file 1 [file 1678-8060-mioc-115-e200184-s.pdf]

TABLE I

Proteins highly druggable (HD) with structures modeled from a template that was co-crystallised in the presence of an inhibitor or a drug-like compound (MD+)

Locus tag	Gene	Gene product	DS
AWH67_RS00420	*thiE*	thiamine phosphate synthase	1
AWH67_RS01825	-------	cytochrome c family protein	1
AWH67_RS04570	-------	acyl-CoA dehydrogenase	1
AWH67_RS01650	*fabI*	enoyl-[acyl-carrier-protein] reductase	0.99
AWH67_RS01730	*infB*	translation initiation factor IF-2	0.99
AWH67_RS01235	*prfC*	peptide chain release factor 3	0.99
AWH67_RS04810	*gcvP*	glycine dehydrogenase (aminomethyl-transferring)	0.99
AWH67_RS01195	-------	LPS export ABC transporter ATP-binding protein	0.98
AWH67_RS04335	*nuoM*	NADH-quinone oxidoreductase subunit M	0.98
AWH67_RS00525	-------	ABC transporter ATP-binding protein	0.98
AWH67_RS02850	*dxs*	1-deoxy-D-xylulose-5-phosphate synthase	0.98
AWH67_RS05345	-------	DUF1009 domain-containing protein	0.98
AWH67_RS02660	*thiE*	thiamine phosphate synthase	0.97
AWH67_RS04105	*folA*	dihydrofolate reductase	0.97
AWH67_RS03780	*lgt*	prolipoprotein diacylglyceryl transferase	0.97
AWH67_RS01010	*phaAB*	monovalent cation/H+ antiporter subunit A	0.97
AWH67_RS03605	-------	ABC transporter substrate-binding protein	0.97
AWH67_RS01025	*lpdA1*	dihydrolipoyl dehydrogenase	0.96
AWH67_RS02300	*serS*	serine--tRNA ligase	0.96
AWH67_RS02640	*thiC*	phosphomethylpyrimidine synthase	0.96
AWH67_RS02270	-------	peptidylprolyl isomerase	0.96
AWH67_RS03970	*murE*	UDP-N-acetylmuramoyl-L-alanyl-D-glutamate--2, 6-diaminopimelate ligase	0.95
AWH67_RS03610	-------	ABC transporter substrate-binding protein	0.95
AWH67_RS02235	*pdhA*	pyruvate dehydrogenase (acetyl-transferring) E1 component subunit alpha	0.95
AWH67_RS03355	*pyrD*	quinone-dependent dihydroorotate dehydrogenase	0.94
AWH67_RS04295	*nuoE*	NADH-quinone oxidoreductase subunit	0.94
AWH67_RS02305	*rlmJ*	23S rRNA (adenine(2030)-N(6))-methyltransferase	0.94
AWH67_RS05465	-------	NADPH:quinone oxidoreductase	0.93
AWH67_RS04150	*cycA*	D-serine/D-alanine/glycine transporter	0.93
AWH67_RS03525	*yidC*	membrane protein insertase	0.92
AWH67_RS05265	-------	penicillin-binding protein	0.92
AWH67_RS02020	*bioY*	biotin transporter	0.91
AWH67_RS02100	*tuf*	elongation factor Tu	0.91
AWH67_RS02505	*panC*	pantoate--beta-alanine ligase	0.91
AWH67_RS05820	*glnA*	type I glutamate--ammonia ligase	0.91
AWH67_RS02795	-------	HAD family phosphatase	0.90
AWH67_RS02025	*pheP*	amino acid permease	0.90
AWH67_RS02215	*lipA*	lipoyl synthase	0.88
AWH67_RS02915	*ispE*	4-(cytidine 5'-diphospho)-2-C-methyl-D-erythritol kinase	0.88
AWH67_RS02820	*ispH*	4-hydroxy-3-methylbut-2-enyl diphosphate reductase	0.88
AWH67_RS01255	*tsaE*	tRNA (adenosine(37)-N6)-threonylcarbamoyltransferase complex ATPase subunit type 1	0.88
AWH67_RS01475	*afeB*	manganese transporter	0.86
AWH67_RS03135	*usp*	universal stress protein	0.86
AWH67_RS00295	*dnaG*	DNA primase	0.86
AWH67_RS02890	*purD*	phosphoribosylamine--glycine ligase	0.86
AWH67_RS05775	-------	MFS transporter	0.86
AWH67_RS04220	-------	MFS transporter	0.86
AWH67_RS00840	-------	DNA-binding protein	0.85
AWH67_RS01270	*folC*	bifunctional folylpolyglutamate synthase/dihydrofolate synthase	0.85
AWH67_RS02790	*ccrMIM*	site-specific DNA-methyltransferase	0.85
AWH67_RS03180	*mgtE*	magnesium transporter	0.85
AWH67_RS05670	*purN*	phosphoribosylglycinamide formyltransferase	0.85
AWH67_RS03740	*ychF*	redox-regulated ATPase	0.84
AWH67_RS01750	*trmB*	tRNA (guanosine(46)-N7)-methyltransferase	0.84
AWH67_RS02770	*ppdK*	pyruvate, phosphate dikinase	0.84
AWH67_RS02665	*thiD*	hydroxymethylpyrimidine/phosphomethylpyrimidine kinase	0.84
AWH67_RS04635	*cobT*	nicotinate-nucleotide--dimethylbenzimidazole phosphoribosyltransferase	0.84

Locus tag	Gene	Gene product	DS
AWH67_RS02805	-------	PAS domain S-box protein	0.84
AWH67_RS02395	*acpP*	acyl carrier protein	0.83
AWH67_RS05400	*gor*	glutathione-disulfide reductase	0.83
AWH67_RS04580	*pyrC*	dihydroorotase	0.83
AWH67_RS00580	*ispG*	4-hydroxy-3-methylbut-2-en-1-yl diphosphate synthase (flavodoxin)	0.83
AWH67_RS00500	*tkt*	transketolase	0.82
AWH67_RS03115	*ugpC*	sn-glycerol-3-phosphate import ATP-binding protein	0.82
AWH67_RS01870	*pstB*	phosphate ABC transporter ATP-binding protein	0.82
AWH67_RS00330	*dut*	dUTP diphosphatase	0.81
AWH67_RS00950	*dac*	D-alanyl-D-alanine carboxypeptidase	0.81
AWH67_RS04025	*ftsZ*	cell division protein	0.81
AWH67_RS05575	-------	MerR family transcriptional regulator	0.81
AWH67_RS04000	*murC*	UDP-N-acetylmuramate--L-alanine ligase	0.81
AWH67_RS02005	*aroP*	amino acid permease	0.80
AWH67_RS01035	*sucA*	2-oxoglutarate dehydrogenase E1 component	0.80
AWH67_RS02680	-------	PAS domain S-box protein	0.80
AWH67_RS04690	*purL*	phosphoribosylformylglycinamidine synthase subunit	0.79
AWH67_RS01600	*acnA*	aconitate hydratase	0.79
AWH67_RS04645	-------	hybrid sensor histidine kinase/response regulator	0.791
AWH67_RS03325	-------	16S rRNA (uracil(1498)-N(3))-methyltransferase	0.79
AWH67_RS03955	*rsmH*	ribosomal RNA small subunit methyltransferase H	0.79
AWH67_RS03575	*etfA*	electron transfer flavoprotein subunit alpha/FixB family protein	0.78
AWH67_RS05895	*secY*	preprotein translocase subunit	0.78
AWH67_RS05420	-------	ATP-dependent helicase	0.78
AWH67_RS01705	-------	class I SAM-dependent methyltransferase	0.78
AWH67_RS01510	*aroA*	3-phosphoshikimate 1-carboxyvinyltransferase	0.78
AWH67_RS03700	-------	N-acetyltransferase	0.77
AWH67_RS03085	-------	ABC transporter ATP-binding protein	0.77
AWH67_RS04890	-------	iron ABC transporter ATP-binding protein	0.77
AWH67_RS03075	*nrdI*	class Ib ribonucleoside-diphosphate reductase assembly flavoprotein	0.76
AWH67_RS05700	*rimL*	50S ribosomal protein L7/L12-serine acetyltransferase	0.76
AWH67_RS04315	*nuoI*	NADH-quinone oxidoreductase subunit I	0.76
AWH67_RS04490	*tmk*	dTMP kinase	0.75
AWH67_RS02160	*trmFO*	FADH(2)-oxidising methylenetetrahydrofolate--tRNA-(uracil(54)-C(5))- methyltransferase	0.746
AWH67_RS05705	*recG*	DNA helicase	0.74
AWH67_RS05740	*argS*	arginine--tRNA ligase	0.74
AWH67_RS01350	-------	radical SAM protein	0.74
AWH67_RS02410	*uppP*	undecaprenyl-diphosphatase	0.74
AWH67_RS04300	*nouF*	NADH-quinone oxidoreductase subunit F	0.73
AWH67_RS03045	*secA*	preprotein translocase subunit	0.73
AWH67_RS04575	*pyrB*	aspartate carbamoyltransferase	0.73
AWH67_RS04705	-------	MFS transporter	0.73
AWH67_RS01215	*rlmN*	23S rRNA (adenine(2503)-C(2))-methyltransferase	0.72
AWH67_RS01935	*tadA*	nucleoside deaminase	0.72
AWH67_RS03730	*trmL*	tRNA (cytidine(34)-2'-O)-methyltransferase	0.72
AWH67_RS04365	*lolD*	ABC transporter ATP-binding protein	0.71
AWH67_RS00495	*gap*	type I glyceraldehyde-3-phosphate dehydrogenase	0.71
AWH67_RS01405	*lepA*	elongation factor 4	0.71
AWH67_RS00720	*sdhC*	succinate dehydrogenase, cytochrome b556 subunit	0.7
AWH67_RS01335	-------	alpha/beta hydrolase	0.7
AWH67_RS02905	*tsaC*	threonylcarbamoyl-AMP synthase	0.7
AWH67_RS03335	*folD*	bifunctional methylenetetrahydrofolate dehydrogenase/methenyltetrahydrofolate cyclohydrolase	0.7
AWH67_RS01755	*metK*	methionine adenosyltransferase	0.7

DS: druggability score.

TABLE II

List of prioritised protein targets of *Bartonella bacilliformis* USM-LMMB07 proteins filtering by druggability scores, impact to gut microbiome and human host

Locus tag	Gene	Gene product	DS	Human off target	Gut microb (%)
AWH67_RS02160	trmFO	FADH(2)-oxidising methylenetetrahydrofolate--tRNA-(uracil(54)-C(5))- methyltransferase	0.75	1	31.86
AWH67_RS01090	*parB*	chromosome partitioning protein	0.82	1	33.19
AWH67_RS05895	*secY*	preprotein translocase subunit	0.78	1	32.30
AWH67_RS03000	-------	aspartate kinase	0.74	1.	30.97
AWH67_RS05565	fabH	3-oxoacyl-ACP synthase III	0.89	1	29.65
AWH67_RS01180	*ihfB*	integration host factor subunit beta	0.76	1	30.09
AWH67_RS05890	*adk*	adenylate kinase	0.78	0.64	27.88
AWH67_RS01565	*argD*	acetylornithine transaminase	0.85	0.65	25.66
AWH67_RS01195	-------	LPS export ABC transporter ATP-binding protein	0.98	0.70	24.78
AWH67_RS04595	*topA*	type I DNA topoisomerase	0.71	0.75	25.66
AWH67_RS03730	*trmL*	tRNA (cytidine(34)-2'-O)-methyltransferase	0.72	1	23.45
AWH67_RS01845	*moeB*	molybdopterin-synthase adenylyltransferase	0.75	0.60	24.34
AWH67_RS02690	-------	DNA-binding response regulator	0.78	1	23.89
AWH67_RS02005	*aroP*	amino acid permease	0.80	0.75	22.57
AWH67_RS04665	purB	adenylosuccinate lyase	0.86	0.73	22.57
AWH67_RS02025	*PheP*	amino acid permease	0.90	0.76	22.12
AWH67_RS04575	*pyrB*	aspartate carbamoyltransferase	0.73	0.65	20.80
AWH67_RS01020	*lys1*	saccharopine dehydrogenase	0.73	1	21.24
AWH67_RS03940	*chvD*	energy-dependent translational throttle protein	0.93	0.72	21.24
AWH67_RS04890	-------	iron ABC transporter ATP-binding protein	0.77	0.70	20.80
AWH67_RS05820	*glnA*	type I glutamate--ammonia ligase	0.91	0.76	19.47
AWH67_RS05315	*cdsA*	phosphatidate cytidylyltransferase	0.90	0.62	18.14
AWH67_RS04685	*PurQ*	phosphoribosylformylglycinamidine synthase subunit	0.89	0.75	17.70
AWH67_RS04690	*purL*	phosphoribosylformylglycinamidine synthase subunit	0.79	0.77	17.26
AWH67_RS05805	ssb	single-stranded DNA-binding protein	0.87	0.65	18.14
AWH67_RS04150	*cycA*	D-serine/D-alanine/glycine transporter	0.93	0.78	16.37
AWH67_RS00340	*exbB*	biopolymer transporter	0.95	1	16.81
AWH67_RS03955	*rsmH*	ribosomal RNA small subunit methyltransferase H	0.79	0.61	15.04
AWH67_RS04550	-------	cold-shock protein	0.73	1	15.93
AWH67_RS02790	*ccrMIM*	site-specific DNA-methyltransferase	0.85	1	15.04
AWH67_RS00880	*lysA*	diaminopimelate decarboxylase	0.96	0.70	15.04
AWH67_RS05705	*recG*	DNA helicase	0.74	0.74	14.16
AWH67_RS05055	*flil*	flagellar protein export ATPase	0.77	0.69	14.60
AWH67_RS02820	*ispH*	4-hydroxy-3-methylbut-2-enyl diphosphate reductase	0.88	1	13.27
AWH67_RS04000	murC	UDP-N-acetylmuramate--L-alanine ligase	0.81	1	12.83
AWH67_RS01215	*rlmN*	23S rRNA (adenine(2503)-C(2))-methyltransferase	0.72	1	13.27
AWH67_RS05270	*clpS*	ATP-dependent Clp protease adapter	0.81	1	12.83
AWH67_RS04450	-------	SUF system Fe-S cluster assembly protein	0.80	1	12.39
AWH67_RS02595	-------	iron ABC transporter permease	0.99	1	11.95
AWH67_RS04880	-------	iron ABC transporter permease	0.84	1	11.95
AWH67_RS03900	*dnaG*	DNA primase	0.77	1	11.95
AWH67_RS03075	*nrdl*	class Ib ribonucleoside-diphosphate reductase assembly flavoprotein	0.76	1	11.06
AWH67_RS01510	*aroA*	3-phosphoshikimate 1-carboxyvinyltransferase	0.78	1	10.18
AWH67_RS01885	-------	aminopeptidase	0.96	1	11.50
AWH67_RS03025	-------	L-cystine transporter	0.95	0.72	11.50
AWH67_RS01985	*mutL*	DNA mismatch repair protein	0.84	0.66	11.50
AWH67_RS04105	*folA*	dihydrofolate reductase	0.97	0.68	9.29
AWH67_RS04490	*tmk*	dTMP kinase	0.75	1	9.29
AWH67_RS01475	*afeB*	manganese transporter	0.86	0.71	10.18
AWH67_RS05335	*fabZ*	beta-hydroxyacyl-ACP dehydratase	0.92	1	9.73
AWH67_RS00805	*htpX*	zinc metalloprotease	0.86	1	10.18
AWH67_RS03935	-------	bifunctional nicotinamidase/pyrazinamidase	0.86	1	9.73
AWH67_RS03645	-------	DegT/DnrJ/EryC1/StrS aminotransferase family protein	0.80	1	10.18
AWH67_RS00395	*rpmE*	50S ribosomal protein L31	0.80	1	10.18
AWH67_RS04140	pdxJ	pyridoxine 5'-phosphate synthase	0.72	1	9.73
AWH67_RS01155	-------	BAX inhibitor (BI)-1/YccA family protein	0.74	0.71	9.73
AWH67_RS02675	*pepN*	aminopeptidase N	0.96	0.75	9.29
AWH67_RS03175	-------	M3 family peptidase	0.98	0.70	9.29

Locus tag	Gene	Gene product	DS	Human off target	Gut microb (%)
AWH67_RS01895	-------	dipeptidase	0.79	0.73	9.29
AWH67_RS00405	-------	inositol monophosphatase	0.73	0.66	8.85
AWH67_RS04095	-------	MFS transporter	0.90	1	8.85
AWH67_RS01650	*fabI*	enoyl-[acyl-carrier-protein] reductase	0.99	0.71	7.08
AWH67_RS05410	*nadE*	NAD+ synthase	0.73	1	8.41
AWH67_RS03545	*pyrF*	orotidine-5'-phosphate decarboxylase	0.88	1	7.52
AWH67_RS02870	fabI	enoyl-[acyl-carrier-protein] reductase	0.77	0.73	7.52
AWH67_RS00435	*ruvC*	crossover junction endodeoxyribonuclease	0.70	1	7.52
AWH67_RS02305	*rmlJ*	23S rRNA (adenine(2030)-N(6))-methyltransferase	0.94	1	5.75
AWH67_RS06240	*fliY*	flagellar motor switch protein	0.75	1	7.08
AWH67_RS05735	-------	deoxyguanosinetriphosphate triphosphohydrolase	0.87	1	6.19
AWH67_RS02550	*rpoZ*	DNA-directed RNA polymerase subunit omega	0.82	1	6.64
AWH67_RS03105	*ugpA*	sn-glycerol-3-phosphate ABC transporter permease	0.96	1	6.19
AWH67_RS06105	*secD*	protein translocase subunit	0.77	1	6.19
AWH67_RS00185	*maf2*	septum formation protein	0.85	0.64	5.31
AWH67_RS02935	-------	polyprenyl synthetase family protein	0.83	0.72	5.75
AWH67_RS04735	dnaE	DNA polymerase III subunit alpha	0.88	1	5.31
AWH67_RS04630	*grp*	Lrp/AsnC family transcriptional regulator	0.84	1	5.31
AWH67_RS05540	*ribH*	6,7-dimethyl-8-ribityllumazine synthase	0.71	1	4.87
AWH67_RS01345	*dapE*	succinyl-diaminopimelate desuccinylase	0.75	0.78	4.87
AWH67_RS02695	*htrA*	PDZ domain-containing protein	0.73	0.66	5.31
AWH67_RS02660	*thiE*	thiamine phosphate synthase	0.97	1	3.54
AWH67_RS00895	-------	TlpA family protein disulfide reductase	0.82	1	4.42
AWH67_RS05575	-------	MerR family transcriptional regulator	0.81	1	3.98
AWH67_RS02030	*gpsA*	glycerol-3-phosphate dehydrogenase (NAD(P)(+))	0.94	0.75	4.87
AWH67_RS02905	*tsaC*	threonylcarbamoyl-AMP synthase	0.70	0.69	3.54
AWH67_RS01945	-------	16S rRNA (guanine(966)-N(2))-methyltransferase	0.98	1	3.98
AWH67_RS03525	*yidC*	membrane protein insertase	0.92	1	3.98
AWH67_RS00440	*ruvA*	Holliday junction branch migration protein	0.77	1	4.42
AWH67_RS05515	*argC*	N-acetyl-gamma-glutamyl-phosphate reductase	0.72	1	3.98
AWH67_RS04335	*nuoM*	NADH-quinone oxidoreductase subunit M	0.98	0.61	3.10
AWH67_RS01720	*truB*	tRNA pseudouridine(55) synthase	0.79	0.67	3.98
AWH67_RS01255	*tsaE*	tRNA (adenosine(37)-N6)-threonylcarbamoyltransferase complex ATPase subunit type 1	0.88	1	3.54
AWH67_RS00580	*ispG*	4-hydroxy-3-methylbut-2-en-1-yl diphosphate synthase (flavodoxin)	0.83	1	3.10
AWH67_RS02995	*nhaA*	Na+/H+ antiporter	0.80	1	3.98
AWH67_RS01890	*mutM*	formamidopyrimidine-DNA glycosylase	0.93	1	3.54
AWH67_RS00210	purK	5-(carboxyamino)imidazole ribonucleotide synthase	0.87	1	3.10
AWH67_RS02680	-------	PAS domain S-box protein	0.80	1	2.65
AWH67_RS05445	-------	heat-shock protein	0.71	1	3.54
AWH67_RS05740	*argS*	arginine--tRNA ligase	0.74	0.67	2.21
AWH67_RS05775	-------	MFS transporter	0.86	1	2.21
AWH67_RS02020	*bioY*	biotin transporter	0.91	1	1.77
AWH67_RS02410	*uppP*	undecaprenyl-diphosphatase	0.74	1	1.33
AWH67_RS00825	-------	carbonic anhydrase	0.84	1	2.21
AWH67_RS06050	*gltJ1*	amino acid ABC transporter permease	0.74	1	2.65
AWH67_RS04320	*nuoJ*	NADH-quinone oxidoreductase subunit J	0.71	1	2.21
AWH67_RS01010	phaAB	monovalent cation/H+ antiporter subunit A	0.97	0.66	2.21
AWH67_RS04050	-------	aminopeptidase P family protein	0.99	0.62	2.65
AWH67_RS02570	-------	transglycosylase	0.90	1	2.21
AWH67_RS02665	*thiD*	hydroxymethylpyrimidine/phosphomethylpyrimidine kinase	0.84	1	0.88
AWH67_RS00135	-------	terminase	0.84	1	2.21
AWH67_RS04340	*nuoN*	NADH-quinone oxidoreductase subunit	1	0.78	1.77
AWH67_RS02405	*fabD*	[acyl-carrier-protein] S-malonyltransferase	0.91	0.68	1.77
AWH67_RS04795	-------	rhodanese domain-containing protein	0.75	0.66	1.77
AWH67_RS03780	*lgt*	prolipoprotein diacylglyceryl transferase	0.97	1	0.88
AWH67_RS01015	*nspC*	carboxynorspermidine decarboxylase	0.90	1	1.33
AWH67_RS03835	-------	TrkH family potassium uptake protein	0.90	1	1.77
AWH67_RS03180	*mgtE*	magnesium transporter	0.85	1	0.88
AWH67_RS00080	-------	hypothetical protein	0.76	1	1.77

Locus tag	Gene	Gene product	DS	Human off target	Gut microb (%)
AWH67_RS05700	*rimL*	50S ribosomal protein L7/L12-serine acetyltransferase	0.76	1	0.88
AWH67_RS01375	-------	DNA-binding response regulator	0.74	1	1.77
AWH67_RS01675	-------	peptidoglycan endopeptidase	0.73	1	1.77
AWH67_RS01270	*folC*	bifunctional folylpolyglutamate synthase/dihydrofolate synthase	0.85	0.70	0.44
AWH67_RS05400	*gor*	glutathione-disulfide reductase	0.83	0.64	0
AWH67_RS03970	murE	UDP-N-acetylmuramoyl-L-alanyl-D-glutamate--2, 6-diaminopimelate ligase	0.95	1	0
AWH67_RS00420	*thiE*	thiamine phosphate synthase	1	1	0
AWH67_RS02270	-------	peptidylprolyl isomerase	0.96	1	0
AWH67_RS00680	-------	efflux RND transporter periplasmic adaptor subunit	0.94	1	1.33
AWH67_RS01415	-------	TIGR00730 family Rossman fold protein	0.93	1	1.33
AWH67_RS02795	-------	HAD family phosphatase	0.90	1	0.44
AWH67_RS02915	*ispE*	4-(cytidine 5'-diphospho)-2-C-methyl-D-erythritol kinase	0.88	1	0
AWH67_RS01750	*trmB*	tRNA (guanosine(46)-N7)-methyltransferase	0.84	1	0
AWH67_RS02925	-------	S49 family peptidase	0.84	1	1.33
AWH67_RS05130	*flhA*	flagellar biosynthesis protein	0.83	1	1.33
AWH67_RS00950	*dac*	D-alanyl-D-alanine carboxypeptidase	0.81	1	0.44
AWH67_RS03665	-------	DNA-binding response regulator	0.80	1	1.33
AWH67_RS03325	-------	16S rRNA (uracil(1498)-N(3))-methyltransferase	0.79	1	0
AWH67_RS01355	*rdgB*	non-canonical purine NTP pyrophosphatase	0.93	0.72	0.44
AWH67_RS04580	*pyrC*	dihydroorotase	0.83	0.75	0.44
AWH67_RS03085	-------	ABC transporter ATP-binding protein	0.77	0.69	0.44
AWH67_RS04205	*lipB*	lipoate-protein ligase B	0.73	0.65	0.88
AWH67_RS00720	*sdhC*	succinate dehydrogenase, cytochrome b556 subunit	0.70	0.60	0
AWH67_RS04570	-------	acyl-CoA dehydrogenase	1	1	0
AWH67_RS05345	-------	DUF1009 domain-containing protein	0.98	1	0
AWH67_RS00535	*thiN*	thiamine diphosphokinase	0.94	1	0.44
AWH67_RS05265	-------	penicillin-binding protein	0.92	1	0
AWH67_RS03135	usp	universal stress protein	0.86	1	0
AWH67_RS04220	-------	MFS transporter	0.86	1	0
AWH67_RS00840	-------	DNA-binding protein	0.85	1	0
AWH67_RS04635	*cobT*	nicotinate-nucleotide--dimethylbenzimidazole phosphoribosyltransferase	0.84	1	0
AWH67_RS01690	-------	asparaginase	0.81	1	0.88
AWH67_RS00750	*xerC*	tyrosine recombinase	0.79	1	0.88
AWH67_RS04645	-------	hybrid sensor histidine kinase/response regulator	0.79	1	0
AWH67_RS01705	-------	class I SAM-dependent methyltransferase	0.78	1	0
AWH67_RS03700	-------	N-acetyltransferase	0.77	1	0
AWH67_RS01745	*rimP*	ribosome maturation factor	0.75	1	0.88
AWH67_RS00525	-------	ABC transporter ATP-binding protein	0.98	0.70	0
AWH67_RS05465	-------	NADPH:quinone oxidoreductase	0.93	0.72	0
AWH67_RS00295	*dnaG*	DNA primase	0.86	0.76	0
AWH67_RS01825	-------	cytochrome c family protein	1	0.62	0
AWH67_RS04560	*gatC*	Asp-tRNA(Asn)/Glu-tRNA(Gln) amidotransferase GatCAB subunit C	0.81	0.70	0.44
AWH67_RS02375	*rsmA*	16S rRNA (adenine(1518)-N(6)/adenine(1519)-N(6))- dimethyltransferase	0.83	0.62	0.44
AWH67_RS01335	-------	alpha/beta hydrolase	0.70	0.67	0
AWH67_RS05475	-------	beta-ketoacyl-ACP synthase II	0.97	1	0
AWH67_RS03605	-------	ABC transporter substrate-binding protein	0.97	1	0
AWH67_RS03610	-------	ABC transporter substrate-binding protein	0.95	1	0
AWH67_RS01975	lpxK	tetraacyldisaccharide 4'-kinase	0.93	1	0
AWH67_RS00715	*sdhD*	succinate dehydrogenase, hydrophobic membrane anchor protein	0.92	1	0
AWH67_RS01150	-------	diguanylate cyclase	0.91	1	0
AWH67_RS01100	*rsmG*	ribosomal RNA small subunit methyltransferase G	0.88	1	0
AWH67_RS03995	*murG*	undecaprenyldiphospho-muramoylpentapeptide beta-N- acetylglucosaminyltransferase	0.73	1	0
AWH67_RS04040	*recN*	DNA repair protein	0.85	1	0.44
AWH67_RS02805	-------	PAS domain S-box protein	0.84	1	0
AWH67_RS01245	*addA*	double-strand break repair helicase	0.82	1	0.44
AWH67_RS01230	*lisS*	lysine--tRNA ligase	0.80	1	0
AWH67_RS05370	-------	amino acid ABC transporter	0.78	1	0.44
AWH67_RS05235	rplJ	50S ribosomal protein L10	0.97	0.77	0.44
AWH67_RS03530	*rnpA*	ribonuclease P protein component	0.74	1	0.44

Locus tag	Gene	Gene product	DS	Human off target	Gut microb (%)
AWH67_RS04440	*sufD*	Fe-S cluster assembly protein	0.73	1	0.44
AWH67_RS01000	*phaD*	monovalent cation/H+ antiporter subunit D	0.97	0.75	0.44
AWH67_RS04985	-------	autotransporter outer membrane beta-barrel domain-containing protein	0.70	1	0.44
AWH67_RS01955	*cysQ*	3'(2'),5'-bisphosphate nucleotidase	0.93	0.76	0
AWH67_RS05195	*hisS*	histidine--tRNA ligase	0.88	0.70	0
AWH67_RS04425	-------	cysteine desulfurase	0.83	0.69	0
AWH67_RS05835	-------	RluA family pseudouridine synthase	0.80	0.68	0
AWH67_RS04705	-------	MFS transporter	0.73	0.70	0
AWH67_RS00130	-------	phage portal protein	0.99	1	0
AWH67_RS03305	-------	efflux RND transporter periplasmic adaptor subunit	0.99	1	0
AWH67_RS00835	-------	glutamine amidotransferase	0.97	1	0
AWH67_RS02360	*lptD*	LPS-assembly protein	0.97	1	0
AWH67_RS02775	*dsbA*	DsbA family protein	0.97	1	0
AWH67_RS02325	-------	autotransporter outer membrane beta-barrel domain-containing protein	0.97	1	0
AWH67_RS05490	-------	hypothetical protein	0.94	1	0
AWH67_RS01120	-------	phosphoenolpyruvate synthase regulatory protein	0.94	1	0
AWH67_RS04870	-------	autotransporter outer membrane beta-barrel domain-containing protein	0.94	1	0
AWH67_RS04995	-------	autotransporter outer membrane beta-barrel domain-containing protein	0.92	1	0
AWH67_RS01550	-------	Smr domain-containing protein	0.91	1	0
AWH67_RS02355	*lptG*	LPS export ABC transporter permease	0.90	1	0
AWH67_RS02015	*ecfT*	energy-coupling factor transporter transmembrane protein	0.90	1	0
AWH67_RS04930	*flgK*	flagellar hook-associated protein	0.87	1	0
AWH67_RS04940	-------	DNA-binding response regulator	0.87	1	0
AWH67_RS03695	-------	metallophosphoesterase	0.86	1	0
AWH67_RS03150	-------	sigma-54-dependent Fis family transcriptional regulator	0.86	1	0
AWH67_RS02200	*ntrY*	PAS domain-containing sensor histidine kinase	0.86	1	0
AWH67_RS01280	-------	cysteine biosynthesis protein	0.85	1	0
AWH67_RS01770	*corC*	HlyC/CorC family transporter	0.85	1	0
AWH67_RS02580	-------	TonB-dependent hemoglobin/transferrin/lactoferrin family receptor	0.83	1	0
AWH67_RS02435	*dnaB*	replicative DNA helicase	0.83	1	0
AWH67_RS00510	*zapA*	cell division protein	0.81	1	0
AWH67_RS00640	-------	hypothetical protein	0.81	1	0
AWH67_RS03710	-------	DUF4159 domain-containing protein	0.81	1	0
AWH67_RS00910	*holA*	DNA polymerase III subunit delta	0.80	1	0
AWH67_RS04990	-------	autotransporter outer membrane beta-barrel domain-containing protein	0.80	1	0
AWH67_RS00980	-------	hypothetical protein	0.79	1	0
AWH67_RS04165	-------	hypothetical protein	0.77	1	0
AWH67_RS03560	-------	pyrroline-5-carboxylate reductase	0.77	1	0
AWH67_RS02350	lptF	LPS export ABC transporter permease	0.77	1	0
AWH67_RS01495	*ndvB*	protein ndvB	0.75	1	0
AWH67_RS05075	*fliM*	flagellar motor switch protein	0.74	1	0
AWH67_RS03870	*tetA*	tetracycline resistance MFS efflux pump	0.98	0.75	0
AWH67_RS02510	*recO*	DNA repair protein	0.73	1	0
AWH67_RS04080	-------	hypothetical protein	0.73	1	0
AWH67_RS00430	-------	DUF1465 domain-containing protein	0.73	1	0
AWH67_RS05000	-------	hypothetical protein	0.72	1	0

DS: druggability score, human off target: this score reflects the results of a blastp search of the pathogen protein in the human proteome database with the scale 1 - max (alignment identity), so when a protein has no hit in the human proteome, the value is 1. Gut microbiome: percentage of the 226 representative microorganisms of human intestinal flora sequenced by the Human Microbiome Project that have at least one hit with any pathogen protein.
